# Current Perspectives on Neonatal Screening for Propionic Acidemia in Japan: An Unexpectedly High Incidence of Patients with Mild Disease Caused by a Common *PCCB* Variant

**DOI:** 10.3390/ijns7030035

**Published:** 2021-06-28

**Authors:** Go Tajima, Reiko Kagawa, Fumiaki Sakura, Akari Nakamura-Utsunomiya, Keiichi Hara, Miori Yuasa, Yuki Hasegawa, Hideo Sasai, Satoshi Okada

**Affiliations:** 1 Division of Neonatal Screening, Research Institute, National Center for Child Health and Development, 2-10-1 Okura, Setagaya-ku, Tokyo 157-8535, Japan; 2 Department of Pediatrics, Hiroshima University Graduate School of Biomedical and Health Sciences, 1-2-3 Kasumi, Minami-ku, Hiroshima 734-8551, Japan; rekagawa@hiroshima-u.ac.jp (R.K.); d185866@hiroshima-u.ac.jp (F.S.); sokada@hiroshima-u.ac.jp (S.O.); 3 Department of Pediatrics, Hiroshima Prefectural Hospital, 1-5-54 Ujinakanda, Minami-ku, Hiroshima 734-8530, Japan; a-utsunomiya@hph.pref.hiroshima.jp; 4 Department of Pediatrics, National Hospital Organization Kure Medical Center and Chugoku Cancer Center, 3-1 Aoyama-cho, Kure 737-0023, Japan; hara.keiichi.dv@mail.hosp.go.jp; 5 Department of Pediatrics, Faculty of Medical Sciences, University of Fukui, 23-3 Matsuoka-Shimoaizuki, Eiheiji-cho, Fukui 910-1193, Japan; miori@u-fukui.ac.jp; 6 Department of Pediatrics, Japanese Red Cross Matsue Hospital, 200 Horomachi, Matsue 690-8506, Japan; yukirin@med.shimane-u.ac.jp; 7 Department of Pediatrics, Graduate School of Medicine, Gifu University, 1-1 Yanagido, Gifu 501-1194, Japan; sasai@gifu-u.ac.jp

**Keywords:** propionic acidemia, tandem mass spectrometry, propionylcarnitine, cardiomyopathy, QT prolongation

## Abstract

Propionic acidemia (PA) is a disorder of organic acid metabolism which typically presents with acute encephalopathy-like symptoms associated with metabolic acidosis and hyperammonemia during the neonatal period. The estimated incidence of symptomatic PA in Japan is 1/400,000. The introduction of neonatal screening using tandem mass spectrometry has revealed a far higher disease frequency of approximately 1/45,000 live births due to a prevalent variant of c.1304T>C (p.Y435C) in *PCCB*, which codes β-subunit of propionyl-CoA carboxylase. Our questionnaire-based follow-up study reveals that most of these patients remain asymptomatic. However, reports on symptomatic patients exhibiting cardiac complications such as cardiomyopathy and QT prolongation have been increasing. Moreover, there were even cases in which these cardiac complications were the only symptoms related to PA. A currently ongoing study is investigating the risk of cardiac complications in patients with neonatal screening-detected PA caused by this common variant.

## 1. Introduction

**Figure 1:**
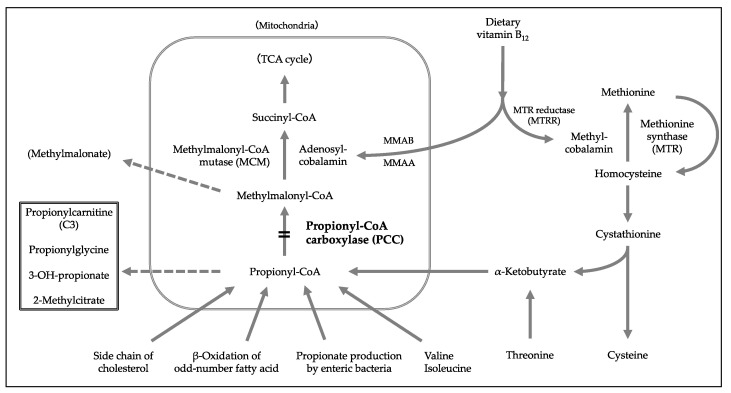
Metabolic pathways related to propionic acidemia. Dashed arrows are alternative pathways leading to disease-specific abnormal metabolites.

Propionic acidemia (PA) is a disorder of organic acid metabolism that results from a congenital deficiency of propionyl-CoA carboxylase (PCC), which is composed of α- and β-subunits, which are coded by *PCCA* (MIM *232000, 13q32.3) and *PCCB* (MIM *232050, 3q22.3), respectively. PCC is located on the catabolic pathway of valine and isoleucine and catalyzes the conversion of propionyl-CoA to methylmalonyl-CoA in the mitochondrial matrix (Figure 1). Deficient PCC activity leads to the accumulation of toxic organic acids such as 3-hydroxypropionate and 2-methylcitrate. Typically, affected patients suffer from an acute acidotic crisis during the neonatal period and present with encephalopathy-like symptoms associated with metabolic acidosis and hyperammonemia immediately after the initiation of lactation, which often leaves neurological sequelae. Similarly, patients with milder phenotypes can show an acute disease onset in infancy or later. Various documented complications of PA include growth and psychomotor retardation, extrapyramidal disorder, cardiac disease, pancreatitis, hearing loss, and optic nerve atrophy. Prevention of disease progression in patients with PA requires a dietary restriction of precursor amino acids (i.e., valine, isoleucine, methionine, threonine, and glycine) accompanied with l-carnitine supplementation. Liver transplantation is considered in patients with poor disease control [1,2].

Aiming at improving the prognosis, several countries list PA as one of the target diseases for neonatal screening, but its utility is not established [1,3]. This review outlines the current perspectives on neonatal screening for PA in Japan.

## 2. Epidemiology in Japan

Before the introduction of neonatal screening using tandem mass spectrometry (MS/MS), the estimated incidence of patients with symptomatic PA was 1/400,000 in Japan. A study previously reported that c.923dupT, c.1196G>A (p.R399Q), and IVS18−6C>G in *PCCA* and c.1228C>T (p.R410W), c.1283C>T (p.T428I), and c.457>C (p.A153P) in *PCCB* were predominant genotypes in symptomatic Japanese patients with PA [4].

A pilot study on MS/MS-based neonatal screening was initiated in 1997 in several areas of the country, and 1,950,000 newborns, corresponding to approximately twice as many live births per year in Japan, were screened throughout the study period. Based on the results, the frequency of PA was 1/45,000, and the unexpectedly high detection rate was due to the presence of c.1304T>C (p.Y435C), a common variant of *PCCB*, with the estimated frequency of heterozygous carriers being 1/86.5 [5]. Homozygotes of the p.Y435C variant were proposed to be classified as those with mildest-type PA as it had not been detected in symptomatic patients.

## 3. Neonatal Screening for PA in Japan: Issues to Be Addressed

After the conclusion of the 15-year pilot study, MS/MS-based neonatal screening was adopted as an official public health service in 2013. The practice of neonatal screening is managed by 60 local public bodies, and the screening tests are assigned to 35 regional laboratories. Propionic acidemia has been included as one of the primary target diseases. Dried blood specimens are usually collected on the fourth or fifth day after birth, as has been done since the introduction of neonatal screening by the Guthrie method in 1977. Though earlier sample collection is thought to be more desirable for MS/MS-based neonatal screening and actually adopted in many countries [6], it has not been approved by the regulatory authority in Japan.

Under this condition, PA and methylmalonic acidemia (MMA) are screened using propionylcarnitine (C3) and the ratio of C3 to acetylcarnitine (C2) as indices. Cutoffs are generally set at 3.6 nmol/mL for C3 and 0.24 for C3/C2. Second-tier tests for C3 and C3/C2-positive dried blood specimens, measuring specific organic acids (3-hidroxypropionate, 2-methylcitrate, methylalonate) and total homocysteine by liquid chromatography-mass spectrometry, can be useful to improve sensitivity and specificity, but they are not available in most laboratories for the lack of official financial support, except in a few laboratories which have the budget for research purpose.

As confirmatory tests for positive subjects, the following biochemical analyses are recommended in the domestic guidelines edited by the Japanese Society for Inherited Metabolic Diseases (JSIMD): organic acids in urine, acylcarnitines, and vitamin B_12_ in serum, amino acids, and total homocysteine in plasma. Results of these biochemical tests are further confirmed by gene panel analysis including *PCCA*, *PCCB*, *MUT*, and genes in the pathway of adenosylcobalamin and methylcobalamin synthesis. Enzymatic assay of neither PCC nor methylmalonyl-CoA mutase (MCM) is supported by the national health insurance system, and they are offered by a few researchers.

Due to strict regulation by the Act on the Protection of Personal Information, it is quite difficult to collect data from each local public body and evaluate them centrally. Japanese Society for Neonatal Screening (JSNS) has collected statistical data on results of neonatal screening tests from each regional laboratory. During the period from 2015 to 2019, 4,715,965 newborns were screened, and the rates of dried blood specimen recollection and close examination for “C3 and C3/C2” were 0.059% and 0.006%, respectively. Though the data did not include detailed information on diagnostic findings of individual patients, positive predictive values for PA and MMA were 30.53% and 9.47%, respectively (partly available at https://www.jsms.gr.jp/contents03-05.html, written in Japanese, accessed on 18 June 2021).

The discrepancy between the frequency of symptomatic patients and that of patients detected by neonatal screening has illustrated that it is unclear what percentage of PA cases detected by neonatal screening is associated with the actual risk of clinical presentation. At the beginning of the pilot study on MS/MS-based neonatal screening, we expected to detect PA patients who were at the risk of presenting with acute metabolic decompensation and/or developing damage in central nervous system. The pilot study and the following official screening program revealed an unexpectedly high frequency of PA due to a common variant *PCCB* p.Y435C. However, it has been difficult to conclude them as false positive subjects, for PCC activities in their lymphocytes were reported to be as low as 3–8% of normal control value [5,7]. Therefore, it is important to determine newborns who will need medical management, which will also reduce the burden of unnecessary anxiety and treatment for patients who will remain asymptomatic throughout life [8]. Further scientific evidence is required to optimize neonatal screening for PA in Japan.

### 3.1. Correlation between Genotypes and Clinical/Biochemical Phenotypes

**Figure 2:**
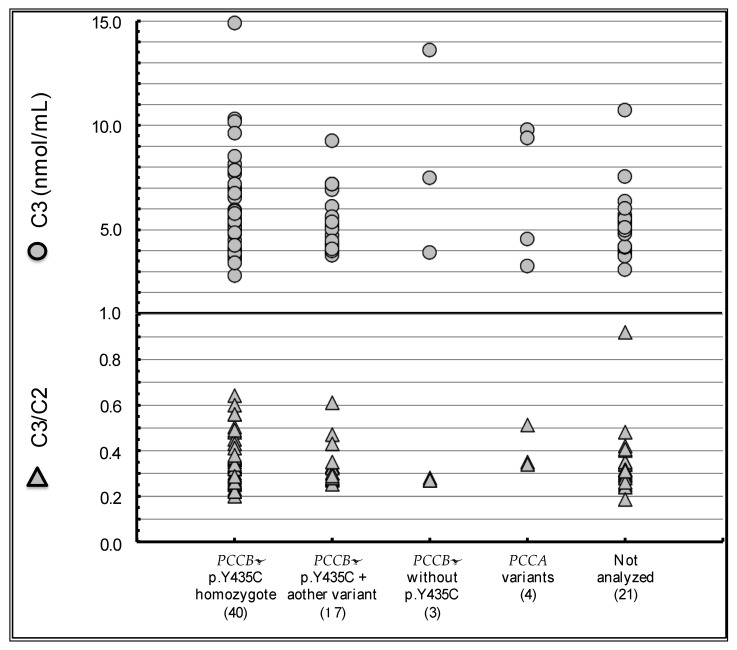
Distributions of propionylcarnitine (C3) and the ratio of C3 to acetylcarnitine (C2) in dried blood specimens of patients detected by neonatal screening. There are no significant differences among the genotypes.

In 2015, we started a countrywide investigation by sending a preliminary questionnaire to 155 hospitals that were responsible for the management of neonatal screening-positive cases in Japan. Responses were collected from 130 hospitals (84%), and 87 patients with PA detected by neonatal screening, and 27 patients with symptomatic PA were identified. The summary of medical information of all 87 patients with neonatal screening-detected PA as well as 15 patients with symptomatic PA is shown in Table 1. Their diagnoses were confirmed by urinary organic acid analysis, using 3-hydroxypropionate, 2-methylcitrate, and propionylglycine as index metabolites. Some of the patients with neonatal screening-detected PA were as old as 20 years of age, and mental retardation was documented in one patient lacking any other PA-associated symptoms. The remaining patients were apparently healthy with no symptoms related to PA. Concerning biochemical examinations, a previous report suggested ketone bodies to be more useful for the evaluation of metabolic stability than the diagnostic metabolites [9]. In our study, mild elevation of total ketone bodies without symptoms was observed in regular examination of 7 neonatal screening-detected patients. Genetic analysis was performed in 72 patients, and the *PCCB* p.Y435C variant was detected in 61 patients, including 41 homozygotes, 16 compound heterozygotes with other *PCCB* variants, and 4 heterozygotes who were not checked for other variants. Six patients who were also checked only for the *PCCB* p.Y435C variant did not harbor it. Biallelic variants of *PCCA* were detected in 5 patients. There were no significant differences in the distributions of C3 and C3/C2 among these genotypes (Figure 2).

Clinical disease onset manifested as acute acidotic crisis during the neonatal and infantile periods in 12 of the 15 patients with symptomatic PA. The remaining three patients had no history of acute metabolic failure, and clues to the diagnosis of PA were mental retardation in 2 patients and syncope in one patient who was subsequently diagnosed with QT prolongation. Genotypes were confirmed in 9 symptomatic patients. Biallelic *PCCB* variants were detected in 4 patients, none of whom harbored the p.Y435C variant. The remaining 5 patients had disease-causing *PCCA* variants.

Among the *PCCB* variants, c.1283C>T (p.T428I) was detected both in patients with neonatal screening-detected PA and in those with symptomatic PA. One patient with symptomatic PA who presented with an acute acidotic crisis during the neonatal period was confirmed to be homozygous for p.T428I, indicating that this variant caused a severe deficiency in PCC enzymatic activity. Five patients with neonatal screening-detected PA were compound heterozygotes for p.T428I and p.Y435C; all of them remained asymptomatic despite minimal medical management consisting of regular physical and biochemical examinations with or without l-carnitine supplementation. These findings suggest that patients with one p.Y435C allele should be expected to be free from the symptoms of PA.

In addition to the abovementioned questionnaire-based study, we have been prospectively collecting the genotypic information of neonatal screening target diseases, including PA, on a larger scale since 2014, based on gene panel analysis using next-generation sequencing with the MiSeq Sequencing System (Illumina, San Diego, CA, USA) performed at the Kazusa DNA Research Institute (Chiba, Japan). The details of the data on *PCCA* and *PCCB* variants are summarized in Table 2. In that cohort, among 58 patients with neonatal screening-detected PA, 31 (53%) were homozygous for p.Y435C, and 17 (29%) were compound heterozygotes for p.Y435C with another variant. In total, 48 of the 58 patients with neonatal screening-detected PA (83%) harbored p.Y435C in at least one allele. None of the 6 symptomatic patients harbored the p.Y435C variant.

### 3.2. Potential Risk of Cardiac Complications in Asymptomatic Patients Detected by Neonatal Screening

Guidelines for the diagnosis and management of PA based on the clinical features of patients studied in large scales were published in 2014 [10] and revised in 2021 [1]. It has become clear that cardiac complications, mainly cardiomyopathy and QT prolongation, are quite specific to PA compared with other similar organic acid disorders and that these complications are primary causes of death during the chronic phase of PA [1,11,12,13,14]. Though precise pathophysiology has not been fully understood, mitochondrial impairment is suggested for the development of cardiomyopathy [2,15], and acute reduction of the repolarizing potassium currents in cardiomyocytes due to toxic metabolites for the prolonged QTc interval [2,16].

In the present study, cardiomyopathy and/or QT prolongation was observed in 6 out of the 15 symptomatic patients (Table 3). Four of these patients with cardiac complications suffered an acute acidotic crisis, whereas the remaining 2 patients did not have a history of acute metabolic failure, indicating that cardiac lesions could develop regardless of the severity of metabolic derangement, as was suggested in previous reports [12,14,17,18].

These findings raise the question of whether apparently healthy patients with neonatal screening-detected PA in Japan can also develop similar cardiac complications. As an additional study started in 2017, we collected data on cardiac ultrasonography and electrocardiography from 45 and 50 patients, respectively, within the cohort of 87 patients with neonatal screening-detected PA. We did not observe abnormalities except cardiomyopathic changes in one patient, whose genotype has not yet been determined. Thus far, cardiac complications have not been reported in any of the patients who are homozygous for p.Y435C. However, it may require the observation of one generation to clarify whether these patients with mildest-type PA may also develop cardiac lesions in a prospective cohort follow-up study.

To address this uncertainty in a shorter time period, we have planned to search for patients with occult PA by measuring 3-hydroxypropionate and 2-methylcitrate levels in urine and C3 levels in serum in patients with cardiomyopathy and/or QT prolongation. Those with abnormal results will be further analyzed for genotyping. Patients with PA detected in this study may benefit from nutritional therapy and l-carnitine supplementation to reduce the accumulation of abnormal metabolites, though the effects of these therapies on the cardiac complications are not proven [1]. Romano et al. reported that liver transplantation improved not only acidotic symptoms but also cardiomyopathy in patients with PA [12]. In the latest guidelines, however, the recommendation of liver transplantation for PA-based cardiomyopathy is still withheld because there have been unfavorable reports on cases that developed cardiomyopathy after liver transplantation as well as reports on successful cases [1].

## 4. Conclusions

Currently implemented neonatal screening for PA in Japan unexpectedly has led to the detection of a high frequency of patients with PA. Most of these patients detected by neonatal screening harbor at least one *PCCB* p.Y435C allele, which appears to retain sufficient enzymatic function to prevent clinical disease onset. Neonatal screening should not burden newborns and their parents with unnecessary anxiety by overdiagnosis or unnecessary treatment; therefore, the clinical significance of this genotype must be clarified.

## Figures and Tables

**Table 1:** Clinical symptoms and genotypes of patients with propionic acidemia in Japan.

	Propionic Acidemia Detected by Neonatal Screening (*n* = 87)		Symptomatic Patients (*n* = 15)	
Clinical phenotypes (*n*)				
	None	86	Acute acidotic crisis, neonatal onset	9
			Acute acidotic crisis, infantile onset	3
	Mental retardation	1	Mental retardation	2
			Syncope	1
Genetic variants (*n*)				
*PCCB*	p.Y435C homozygote	41	p.T428I homozygote	1
	p.Y435C + p.T428I	5	p.T428I + frameshift variant	1
	p.Y435C + another variant	11	Other biallelic variants	2
	p.Y435C heterozygote ^1^	4	p.Y435C detected	0
	p.Y435C not detected ^1^	6		
*PCCA*	Biallelic variants detected	5	Biallelic variants detected	5
No information of genotypes		15	No information of genotypes	6

^1^ Variants other than *PCCB* p.Y435C were not analyzed.

**Table 2:** Frequencies of major variants in the larger-scale prospective study.

Patients	Gene and Variant	Allele Frequency
Patients detected by neonatal screening (*n* = 58)	*PCCB* p.Y435C	79/116 (68.1%)
	*PCCB* p.T428I	7/116 (6.0%)
	*PCCB* p.I430L	3/116 (2.6%)
	*PCCB* p.S510del	2/116 (1.7%)
Symptomatic patients (*n* = 6)	*PCCA* p.L308fs	2/11 (18.2%)
	*PCCA* p.W559L	2/11 (18.2%)

**Table 3:** Cardiac complications observed in symptomatic patients.

Clinical Phenotype	Number of Patients	Cardiac Complication	Cardiac Findings
Acute acidotic crisis, neonatal onset	9	3	Cardiomyopathy + ventricular tachycardia
			Left ventricular dilatation + QT prolongation
			Mild tricuspid regurgitation
Acute acidotic crisis, infantile onset	3	2	Left ventricular dilatation + mild mitral regurgitation
			QT prolongation
Chronic symptoms only	3	2	QT prolongation
			QT prolongation with syncope
Total	15	7	

## Data Availability

The data presented in this study are available on request from the corresponding author.

## References

[1] Forny P., Höster F., Ballhausen D., Chakrapani A., Chapman K.A., Dionisi-Vici C., Dixon M., Grünert S.C., Grunewald S., Haliloglu G. (2021). Guidelines for the diagnosis and management of methylmalonic acidaemia and propionic acidaemia: First revision. J. Inherit. Metab. Dis..

[2] Haijes H.A., Jans J.J.M., Tas S.Y., Verhoeven-Duif N.M., van Hasselt P.M. (2019). Pathophysiology of propionic and methylmalonic acidemias. Part 1: Complications. J. Inherit. Metab. Dis..

[3] Haijes H.A., van Hasselt P.M., Jans J.J.M., Tas S.Y., Verhoeven-Duif N.M. (2019). Pathophysiology of propionic and methylmalonic acidemias. Part 2: Treatment strategies. J. Inherit. Metab. Dis..

[4] Yang X., Sakamoto O., Matsubara Y., Kure S., Suzuki Y., Aoki Y., Yamaguchi S., Takahashi Y., Nishikubo T., Kawaguchi C. (2004). Mutation spectrum of the *PCCA* and *PCCB* genes in Japanese patients with propionic acidemia. Mol. Genet. Metab..

[5] Yorifuji T., Kawai M., Muroi J., Mamada M., Kurokawa K., Shigematsu Y., Hirano S., Sakura N., Yoshida I., Kuhara T. (2002). Unexpectedly high prevalence of the mild form of propionic acidemia in Japan: Presence of a common mutation and possible clinical implications. Hum. Genet..

[6] Heringer J., Valayannopoulos V., Lund A.M., Wijburg F.A., Freisinger P., Barić I., Baumgartner M.R., Burgard P., Burlina A.B., Chapman K.A. (2016). Impact of age at onset and newborn screening on outcome in organic acidurias. J. Inherit. Metab. Dis..

[7] Gotoh K., Nakajima Y., Tajima G., Watanabe Y., Hotta Y., Kataoka T., Kawade Y., Sugiyama N., Ito T., Kimura K. (2017). Determination of methylmalonyl coenzyme A by ultra high-performance liquid chromatography tandem mass spectrometry for measuring propionyl coenzyme A carboxylase activity in patients with propionic acidemia. J. Chromatogr. B Anal. Technol. Biomed. Life Sci..

[8] Haijes H.A., Molema F., Langeveld M., Janssen M.C., Bosch A.M., van Spronsen F., Mulder M.F., Verhoeven-Duif N.M., Jans J.J.M., van der Ploeg A.T. (2020). Retrospective evaluation of the Dutch pre-newborn screening cohort for propionic acidemia and isolated methylmalonic acidemia: What to aim, expect, and evaluate from newborn screening?. J. Inherit. Metab. Dis..

[9] Haijes H.S., Jans J.J.M., van der Ham M., van Hasselt P.M., Verhoeven-Duif N.M. (2020). Understanding acute metabolic decompensateion in propionic and methylalonic acidemias: A deep metabolic phenotyping approach. Orphanet J. Rare Dis..

[10] Baumgartner M.R., Hörster F., Dionisi-Vici C., Haliloglu G., Karall D., Chapman K.A., Huemer M., Hochuli M., Assoun M., Ballhausen D. (2014). Proposed guidelines for the diagnosis and management of methylmalonic and propionic acidemia. Orphanet J. Rare Dis..

[11] Pena L., Burton B.K. (2012). Survey of health status and complications among propionic acidemia patients. Am. J. Med. Genet. A.

[12] Romano S., Valayannopoulos V., Touati G., Jais J.P., Rabier D., de Keyzer Y., Bonnet D., de Lonlay P. (2010). Cardiomyopathies in propionic aciduria are reversible after liver transplantation. J. Pediatr..

[13] Kölker S., Valayannopoulos V., Burlina A.B., Sykut-Cegielska J., Wijburg F.A., Teles E.L., Zeman J., Dionisi-Vici C., Barić I., Karall D. (2015). The phenotypic spectrum of organic acidurias and urea cycle disorders. Part 2. The evolving clinical phenotype. J. Inherit. Metab. Dis..

[14] Baumgartner D., Scholl-Bürgi S., Sass J.O., Sperl W., Schweigmann U., Stein J.I., Karall D. (2007). Prolonged QTc intervals and decreased left ventricular contractility in patients with propionic acidemia. J. Pediatr..

[15] Baruteau J., Hargreaves I., Krywawych S., Chalasani A., Land J.M., Davison J.E., Kwok M.K., Christov G., Karimova A., Ashworth M. (2014). Successful reversal of propionic acidaemia associated cardiomyopathy: Evidence for low myocardial coenzyme Q10 status and secondary mitochondrial dysfunction as an underlying pathophysiological mechanism. Mitochondrion.

[16] Bodi I., Grünert S.C., Becker N., Stoelzle-Feix S., Spiekerkoetter U., Zehender M., Bugger H., Bode C., Odening K.E. (2016). Mechanisms of acquired long QT syndrome in patients with propionic academia. Heart Rhythm.

[17] Riemersma M., Hazebroek M.R., Helderman-van den Enden A.T.J.M., Salomons G.S., Ferdinandusse S., Brouwers M.C.G., van der Ploeg L., Heymans S., Glatz J.F.C., van den Wijngaard A. (2017). Propionic acidemia as a cause of adult-onset dilated cardiomyopathy. Eur. J. Hum. Genet..

[18] Kölker S., Cazorla A.G., Valayannopoulos V., Lund A.M., Burlina A.B., Sykut-Cegielska J., Wijburg F.A., Teles E.L., Zeman J., Dionisi-Vici C. (2015). The phenotypic spectrum of organic acidurias and urea cycle disorders. Part 1: The initial presentation. J. Inherit. Metab. Dis..

